# Supporting social-emotional competence in pre-service teachers: evidence from higher learning teacher education institutions in Tanzania

**DOI:** 10.3389/fpsyg.2026.1891678

**Published:** 2026-08-07

**Authors:** Patrick Clement Mwananyama

**Affiliations:** Faculty of Education, Mwalimu Nyerere Memorial Academy, Dar es Salaam, Tanzania

**Keywords:** faculty and peer supports, pre-service teacher education, pre-service teachers beliefs, social-emotional competence (SEC), social-emotional learning (SEL)

## Abstract

Social-emotional competence (SEC) is important for teachers' professional practice and wellbeing, yet evidence regarding its development in Tanzanian pre-service teacher education remains limited. Guided by Social Cognitive Theory, this study examined the associations of perceived university curriculum quality, faculty support, and peer support with pre-service teachers' SEC and whether beliefs in social-emotional learning (SEL) accounted statistically for part of these associations. A quantitative cross-sectional survey was conducted among second- and third-year pre-service teachers from two Tanzanian higher education institutions. Of 487 completed questionnaires, 22 were excluded because of highly repetitive response patterns, resulting in a final analytic sample of 465 participants. Confirmatory factor analysis and structural equation modeling were conducted, and indirect associations were examined using 2,000 bootstrap resamples. Curriculum quality, faculty support, and peer support were positively associated with beliefs in SEL (β = 0.19, 0.39, and 0.29, respectively) and directly associated with SEC (β = 0.20, 0.24, and 0.20, respectively; all *p* ≤ 0.002). Beliefs in SEL were positively associated with SEC (β = 0.42, *p* < 0.001). Curriculum quality (β = 0.08), faculty support (β = 0.16), and peer support (β = 0.12) also showed significant indirect associations with SEC through beliefs in SEL, while the corresponding direct associations remained significant. The model accounted for 63.9% of the variance in beliefs in SEL and 90.6% of the variance in SEC. The high intercorrelations among the environmental factors and the large explained variance require cautious interpretation. The findings provide a context-specific application and modest refinement of Social Cognitive Theory by distinguishing curriculum, faculty, and peer support as complementary environmental pathways and identifying beliefs in SEL as a potential domain-specific cognitive mechanism. A preliminary implementation framework is proposed for future evaluation. Because the design was cross-sectional and relied on self-report, temporal and causal relationships cannot be established.

## 1 Introduction

In recent years, social-emotional learning (SEL) has gained growing recognition as a critical component of effective education, not only for students but also for teachers (Greenberg, 2023). The development of social-emotional competence (SEC) among pre-service teachers is fundamental to fostering positive teacher-student relationships, promoting emotional wellbeing, and enhancing academic outcomes in the classroom (Jennings and Greenberg, 2009). As future educators, pre-service teachers must be equipped with the ability to manage emotions, build healthy relationships, and make responsible decisions skills that are essential for personal growth and professional effectiveness (Darling-Hammond and Bransford, 2005; Utami et al., 2025). However, the extent to which teacher education programs foster these competencies remains an area of concern, particularly in Tanzania, where teacher education is undergoing significant reform.

The present study focuses on social-emotional competence (SEC) among pre-service teachers. SEC is related to, but distinct from, social-emotional learning (SEL), emotional intelligence, and teacher wellbeing. SEL refers to the educational processes through which social and emotional capacities are developed and applied (Collaborative for Academic, Social, and Emotional, Learning, 2020; Sun and You, 2026). Emotional intelligence is a broader family of ability- or trait-based theories concerning the perception, understanding, use, and regulation of emotion. Teacher wellbeing concerns teachers' positive functioning and experiences, such as occupational satisfaction, vitality, and manageable stress (Markowitz et al., 2018). In this study, SEC is treated as the competence outcome, SEL as the developmental and instructional domain, and beliefs in SEL as a cognitive-attitudinal orientation toward the value and feasibility of SEL.

For conceptual consistency, this study adopts a CASEL-aligned definition of SEC. Pre-service teachers' SEC is defined as their perceived capacity to understand and regulate themselves, understand and respond to others, maintain constructive professional relationships, and make responsible decisions in teaching-related situations. Five dimensions are included: self-awareness, self-management, social awareness, relationship skills, and responsible decision-making. The teacher-specific interpretation of these dimensions is also informed by the prosocial classroom model, which links teachers' SEC and wellbeing to classroom management, teacher-student relationships, and a supportive classroom climate (Jennings and Greenberg, 2009; Elias and Moceri, 2012; Soutter, 2023; Herman et al., 2025).

In Tanzania the University-based teacher education operates within a competence-based orientation that combines disciplinary study, education courses, teaching methods, and practical teacher training. Recent national guidance for university teacher-education programmes emphasizes interactive and collaborative learning, real-world application, inclusive practice, structured teaching practice, university-school partnerships, mentoring, feedback, reflection, classroom management, professionalism, and positive learning environments (Tanzania Commission for Universities, 2024). These priorities overlap substantially with SEC, even though they are not organized as a single, explicit five-domain SEL framework. This creates an opportunity to integrate SEC into existing curriculum, practicum, assessment, and student-support structures rather than treating SEL as an unrelated add-on.

Cultural relevance is equally important. Community-based research in Mtwara has shown that locally valued social-emotional competencies may place comparatively strong emphasis on respect, social responsibility, obedience, attentive listening, and relational conduct, while also valuing individual qualities such as curiosity, self-direction, and self-belief (Jukes et al., 2018, 2021). These findings should not be generalized mechanically from children or one region to all Tanzanian university students. They nevertheless demonstrate that imported SEC frameworks require cultural examination and that competence may be interpreted through relational obligations and community expectations as well as individual self-regulation.

Institutional implementation also depends on the quality of support surrounding teaching practice. Tanzanian research has identified weaknesses in coordination among pre-service teachers, school mentors, and college or university supervisors, including limited social and instructional support and insufficient formative feedback (Mpate et al., 2021; Shaffy and Ndijuye, 2025). Such challenges are directly relevant to SEC development because opportunities for modeling, guided reflection, emotionally safe feedback, and supervised practice depend on the functioning of these relationships.

The quality of the university curriculum (QUC) plays a foundational role in shaping pre-service teachers' social-emotional development. Curricula that embed SEL principles can help trainees acquire the knowledge, attitudes, and behaviors needed to model and apply emotional intelligence in teaching (Zins et al., 2004). Faculty support (FAS) is equally important, as instructors serve as mentors and emotional guides who model and reinforce social-emotional skills (Durlak et al., 2015). Similarly, peer support (PEST) contributes significantly through collaboration, shared reflection, and emotional exchange, providing pre-service teachers with opportunities to learn and practice social-emotional skills in authentic contexts.

Although international literature increasingly links teachers' SEC with classroom climate, instructional effectiveness, teacher wellbeing, and student outcomes, evidence from Sub-Saharan African teacher-education institutions remains comparatively limited. Existing studies often focus on school pupils, in-service teachers, or intervention outcomes, while less attention has been given to how the institutional and interpersonal environment of teacher preparation is associated with pre-service teachers' own SEC. In particular, limited evidence is available on whether beliefs in SEL help explain the associations of curriculum quality, faculty support, and peer support with SEC.

This study addresses that gap in two ways. First, it tests a theoretically specified model linking three environmental supports including quality of the curriculum (QUC), faculty support (FAS), and peer support (PEST) to SEC, both directly and indirectly through beliefs in SEL. Second, it uses the empirical findings as one input, alongside Tanzanian policy and prior literature, to formulate a preliminary implementation framework. The study carefully distinguishes what is supported by the cross-sectional SEM from what remains a theoretical or programmatic recommendation.

### 1.1 Research questions

1. How perceived university curriculum quality is associated with pre-service teachers' SEC, and is this association statistically indirect through their beliefs in SEL?
2. How faculty support is associated with pre-service teachers' SEC, and is this association statistically indirect through their beliefs in SEL?
3. How peer support is associated with pre-service teachers' SEC, and is this association statistically indirect through their beliefs in SEL?

### 1.2 Theoretical framework

#### 1.2.1 Social cognitive theory (SCT)

This study was grounded in Social Cognitive Theory (SCT), proposed by Albert Bandura, which emphasizes the role of observational learning, imitation, and modeling in the development of behavior, skills, and cognitive patterns (Bandura, 1986). SCT posits that individuals acquire knowledge and competencies not only through direct experiences but also by observing others in their environment (Bandura, 1986). This framework is particularly relevant for understanding how pre-service teachers develop social-emotional competencies by interacting with and observing their peers, faculty, and the university curriculum. Observational learning occurs when individuals learn by watching the behaviors of others and the outcomes of those behaviors (Bandura, 1986). In teacher education, faculty and peers act as role models, demonstrating emotional regulation, conflict resolution, and empathy-building practices. The curriculum can further reinforce modeled behaviors of effective teaching and social-emotional competence. Faculty member who exhibit high levels of socio-emotional skills and provide support serve as primary models for pre-service teachers to emulate. Another key SCT concept is self-efficacy, defined as individuals' belief in their ability to perform specific tasks or behaviors (Bandura, 1997). Pre-service teachers' belief in their capacity to implement SEL effectively shapes their social-emotional competence. Incorporating PTBSEL as a mediating factor aligns with Bandura's assertion that beliefs about capabilities influence actions, reinforcing the development of social-emotional skills.

### 1.3 Hypothesis development

#### 1.3.1 Perceived quality of the university curriculum, beliefs in SEL, and social-emotional competence

The university curriculum can support pre-service teachers' social-emotional development through its content, instructional strategies, and assessment practices. Integrating SEL-related content, including self-awareness, emotional regulation, empathy, interpersonal relationships, and responsible decision-making, may strengthen pre-service teachers' understanding of SEL and its relevance to teaching (Denham and Brown, 2010; Lam and Wong, 2017; Katz et al., 2020).

Experiential and participatory approaches, such as role-playing, cooperative learning, case analysis, reflection, and group discussion, also provide opportunities to practice communication, empathy, emotional awareness, and interpersonal problem-solving (Schonert-Reichl, 2017; Bhardwaj et al., 2025). In addition, assessment practices such as self-reflection, peer assessment, portfolios, and reflective journals may help pre-service teachers monitor their social-emotional development (Farrington et al., 2012; Jennings and Greenberg, 2009).

Consistent with the CASEL framework, a curriculum that incorporates SEL content, active learning, and relevant assessment may be associated with stronger beliefs in SEL and higher social-emotional competence.

**H1a:** Perceived quality of the university curriculum is positively associated with pre-service teachers' beliefs in social-emotional learning.

**H2a:** Perceived quality of the university curriculum is positively associated with pre-service teachers' social-emotional competence.

#### 1.3.2 Faculty support, beliefs in SEL, and social-emotional competence

Faculty support may promote pre-service teachers' social-emotional development through emotional encouragement, academic guidance, mentorship, constructive feedback, and the modeling of appropriate social-emotional behavior. Supportive faculty members can create caring and psychologically safe learning environments that strengthen confidence, resilience, emotional awareness, and interpersonal competence.

Faculty members who demonstrate empathy, patience, and effective emotional regulation also provide observable examples of how social-emotional competencies can be applied in educational settings (Jennings and Greenberg, 2009). Through reflective dialogue, feedback, and professional guidance, they may further help pre-service teachers understand the value of SEL and develop relevant skills for future classroom practice.

From a social cognitive perspective, faculty support represents an important environmental factor that may shape beliefs in SEL while providing opportunities to observe and practice social-emotional competencies. Faculty support is therefore expected to be positively associated with both beliefs in SEL and social-emotional competence.

**H1b:** Faculty support is positively associated with pre-service teachers' beliefs in social-emotional learning.

**H2b:** Faculty support is positively associated with pre-service teachers' social-emotional competence.

#### 1.3.3 Peer support, beliefs in SEL, and social-emotional competence

Peer support provides pre-service teachers with opportunities to exchange ideas, receive encouragement, manage academic challenges, and practice communication, collaboration, empathy, and emotional regulation. Supportive peer relationships may also strengthen belonging, resilience, and psychological wellbeing (Cowie and Wallace, 2000; Lavy and Naama-Ghanayim, 2020).

Collaborative learning allows pre-service teachers to discuss teaching experiences, reflect together, and develop teamwork and interpersonal skills required for effective teaching (Darling-Hammond and Bransford, 2005). These experiences may also demonstrate the practical value of SEL and strengthen beliefs in its importance. Peer support is therefore expected to be positively associated with both beliefs in SEL and social-emotional competence.

**H1c:** Peer support is positively associated with pre-service teachers' beliefs in social-emotional learning.

**H2c:** Peer support is positively associated with pre-service teachers' social-emotional competence.

#### 1.3.4 Pre-service teachers' beliefs in SEL and social-emotional competence

Pre-service teachers' beliefs in SEL concern the extent to which they regard social-emotional learning as important, useful, and relevant to teaching. These beliefs may shape their willingness to engage with SEL-related content, participate in reflective activities, and develop competencies such as self-awareness, emotional regulation, empathy, and relationship management.

Teachers who value SEL may be more receptive to learning opportunities provided through the curriculum, faculty guidance, and peer interaction. Previous research has linked teachers' beliefs with their willingness to adopt and implement SEL-related practices (Weissberg et al., 2015; O'Conner et al., 2017). Beliefs in SEL are therefore expected to be positively associated with social-emotional competence.

**H3:** Pre-service teachers' beliefs in social-emotional learning are positively associated with their social-emotional competence.

#### 1.3.5 The indirect role of pre-service teachers' beliefs in SEL

Social cognitive theory proposes that development reflects relationships among environmental conditions, personal cognitive factors, and behavioral outcomes. In this study, perceived curriculum quality, faculty support, and peer support represent environmental factors; beliefs in SEL represent a personal cognitive factor; and social-emotional competence represents the focal outcome.

A supportive curriculum may strengthen pre-service teachers' understanding of and beliefs in SEL through relevant content, experiential learning, and assessment. Faculty members may reinforce these beliefs through encouragement, feedback, and the modeling of empathy and emotional regulation. Similarly, supportive peer relationships may demonstrate the value of collaboration, emotional support, and constructive communication.

Stronger beliefs in SEL may, in turn, be associated with greater engagement in experiences that support social-emotional development. Beliefs in SEL may therefore statistically account for part of the associations between curriculum quality, faculty support, peer support, and social-emotional competence. Because the data are cross-sectional, these indirect associations should not be interpreted as evidence of temporal or causal mediation.

**H4a:** Pre-service teachers' beliefs in social-emotional learning statistically mediate the association between perceived quality of the university curriculum and social-emotional competence.

**H4b:** Pre-service teachers' beliefs in social-emotional learning statistically mediate the association between faculty support and social-emotional competence.

**H4c:** Pre-service teachers' beliefs in social-emotional learning statistically mediate the association between peer support and social-emotional competence.

### 1.4 Study focus and research gap

Existing research indicates that curriculum quality, faculty support, peer support, and beliefs in SEL may be important for the social-emotional development of pre-service teachers. However, limited evidence is available concerning how these factors are jointly associated with pre-service teachers' social-emotional competence within teacher education institutions, particularly in the Tanzanian higher education context. It also remains unclear whether beliefs in SEL statistically account for part of the associations between institutional and interpersonal support factors and social-emotional competence.

Guided by Social Cognitive Theory, this study therefore examines the relationships among perceived quality of the university curriculum, faculty support, peer support, pre-service teachers' beliefs in SEL, and pre-service teachers' social-emotional competence. It specifically investigates both the direct associations among the variables and the hypothesized indirect associations through beliefs in SEL.

The specific objectives of the study are:

1. To examine the associations of perceived quality of the university curriculum, faculty support, and peer support with pre-service teachers' beliefs in social-emotional learning.
2. To examine the direct associations of perceived quality of the university curriculum, faculty support, and peer support with pre-service teachers' social-emotional competence.
3. To examine the association between pre-service teachers' beliefs in social-emotional learning and their social-emotional competence.
4. To determine whether pre-service teachers' beliefs in social-emotional learning statistically mediate the associations of perceived quality of the university curriculum, faculty support, and peer support with social-emotional competence.

## 2 Materials and methods

### 2.1 Study design, setting, and participants

This study employed a quantitative cross-sectional survey design. Data were collected from second- and third-year pre-service teachers enrolled at St. Augustine University of Tanzania, Mwanza, and Mwalimu Nyerere Memorial Academy, Kivukoni Campus, Dar es Salaam. A simple random sampling technique was used. A sampling frame containing the names and identification numbers of all eligible students was obtained from the participating institutions. Excel-generated random numbers were then used to select 487 participants, giving each eligible student an equal probability of selection.

Before the main study, the questionnaire was pilot-tested with 40 pre-service teachers who had characteristics comparable to those of the main participants but were not included in the final sample. The pilot study assessed item clarity, questionnaire administration, completion time, and preliminary reliability. Following the pilot, several revisions were made. Items identified by participants as unclear, repetitive, or difficult to interpret were reworded using simpler and more direct language. Double-barrelled statements were separated or revised to assess only one idea, and technical terms were replaced with expressions familiar to pre-service teachers. A pilot sample of 40 was considered adequate for preliminary questionnaire reliability testing (Bujang et al., 2024).

Before completing the questionnaire, participants received information about the purpose and procedures of the study and provided informed consent. They were informed that participation was voluntary, responses would remain confidential, and withdrawal was permitted at any time without penalty. No personal identifiers were included in the survey dataset. Questionnaire completion took approximately 8–15 min.

A total of 487 questionnaires were completed. The data were screened for missing values, outliers, normality, and inattentive response patterns. Twenty-two responses were excluded because at least 95% of the substantive items contained the same response option, indicating potential straightlining or insufficient-effort responding (Olbrich et al., 2026). The final analytic sample therefore comprised 465 participants.

### 2.2 Measures

The questionnaire consisted of two sections. The first section collected participants' demographic information, including gender, age, year of study, programme of study, and institution. The second section comprised 53 items measuring five constructs: pre-service teachers' social-emotional competence, perceived quality of the university curriculum, faculty support, peer support, and pre-service teachers' beliefs in social-emotional learning. All items were rated on a five-point Likert scale ranging from 1 (*strongly disagree*) to 5 (*strongly agree*), with higher scores indicating higher levels of the respective constructs. Negatively worded items were reverse-coded before analysis. The instruments were adapted from established measures and relevant conceptual frameworks (Brackett et al., 2012; Collaborative for Academic, Social, and Emotional, Learning, 2017; Grazzani et al., 2024; Torsheim et al., 2000).

#### 2.2.1 Alignment of the measures with the conceptual framework

The selected measures operationalised the environmental, cognitive, and competence components of Social Cognitive Theory (Bandura, 1986). Perceived quality of the university curriculum, faculty support, and peer support represented environmental influences through which social-emotional skills may be taught, modeled, reinforced, and practiced. Pre-service teachers' beliefs in social-emotional learning represented the personal cognitive factor proposed to mediate the relationships between these environmental influences and social-emotional competence. Social-emotional competence was treated as the principal outcome and conceptualized according to the five CASEL dimensions of self-awareness, self-management, social awareness, relationship skills, and responsible decision-making. The items were adapted from the Social-Emotional Competence Teacher Rating Scale, CASEL's *Personal Assessment and Reflection-SEL Competencies for School Leaders, Staff, and Adults*, the Teacher SEL Beliefs Scale, and the teacher- and classmate-support subscales (Brackett et al., 2012; Collaborative for Academic, Social, and Emotional, Learning, 2017; Grazzani et al., 2024; Torsheim et al., 2000). Thus, Social Cognitive Theory explained the proposed relationships among the constructs, while the CASEL framework and the selected instruments defined and measured their substantive dimensions.

#### 2.2.2 Pre-service teachers' SEC

SEC was assessed by 25 items with five dimensions including self-awareness, self-regulation, social-awareness, teacher-student relationship skills, and responsible decision making. The 25 items were adopted and modified from SECTRS-14 (Social-Emotional Competence Teacher Rating Scale with Cronbach's Alpha ranging from 0.625 to 0.828 (Grazzani et al., 2024) and “Personal Assessment and Reflection Tool—SEL Competences for School Leaders, Staff and Adults” from Collaborative for Academic, Social, and Emotional Learning (2019). One example of the item from this section of the survey is “I nearly always stay calm when a student upsets me.” Pre-service teachers were required to answer all items based on a five-point Likert scale: strongly disagree, disagree, neutral, agree, and strongly agree.

#### 2.2.3 Quality of university curriculum

To measure this variable nine items were adopted and modified to fit the context of pre-service teachers from the Personal Assessment and Reflection Tool—SEL Competences for School Leaders, Staff and Adults” from Collaborative for Academic, Social, and Emotional Learning (2019). The modified items in Likert scale of five points required pre-service teachers to indicate the level of influence on pre-service teachers' social-emotional competence. For example of the modified item; “The curriculum content provides opportunity to acquire self-awareness and self-regulation skills.”

#### 2.2.4 Faculty support

The focused on how faculty support influences pre-service teachers' social-emotional competencies was measured based on six items in the form of a Likert scale. The researcher constructed the measuring items from the teacher support sub-scale by Torsheim et al. (2000) which has a Cronbach's Alpha of 0.77. The teacher support sub-scale contains four items which were adopted and modified for example the origin item, “Our teacher treats us fairly” was rated based on a five-Likert scale “Strongly agree” to “strongly disagree” was modified to “Our faculty members treat us fairly.” Similarly, the two items were constructed by the researcher based on related literature.

#### 2.2.5 Peer-support

Based on this variable, pre-service teachers asked to rate the influence of peer-support on their social-emotional competence based on six item scale constructed by the researcher. The four items were taken directly from classmate support sub scale with five points Likert scale ranging from “Always” to “never.” The Likert scale has a Cronbach's Alpha 0.75. Example of one item taken from classmate support “When a classmate is upset, other students comfort him” (Torsheim et al., 2000). Two items were constructed by the researcher to make a total of six items. For example, “Interaction with my peers contributes to my self-awareness and understanding of my social-emotional strengths and challenges.”

#### 2.2.6 Pre-service teachers' belief in social-emotional learning

The study focused on pre-service teachers' belief in SEL. Under this aspect, pre-service teachers were asked to show the level of influence of this factor on pre-service teachers' social-emotional competence based on seven items in the form of a Likert scale that were modified from The Teacher SEL Beliefs Scale (Brackett et al., 2012). The Teacher SEL Belief Scale is a 12-item scale that assesses teachers' comfort with, commitment to, and school culture surrounding SEL. The Commitment subscale measures teachers' interest in improving their SEL competencies. For example, I want to improve my ability to learn social and emotional skills. The comfort subscale measures teachers' perceived confidence and comfort in their abilities to implement SEL. For example, “Taking care of my students' social and emotional needs comes naturally to me.” Finally, the culture subscale measures teachers' perceptions of support and promotion for SEL from their principal and school. For example, “The culture in my school supports the development of children's social and emotional skills.” In Brackett et al. (2012) initial validation sample of 935 K-8 teachers in the USA, these three scales were found to have adequate internal consistency (Cronbach's α ranging from 0.74 to 0.82) and validity. The researcher selected only seven items as they relate to the Tanzania context of the study, and the other five items were excluded.

### 2.3 Data analysis

Data were analyzed using IBM SPSS Statistics version 29 and IBM SPSS Amos version 26. SPSS was used for data screening, descriptive statistics, Pearson correlation analysis, and assessment of internal consistency. Cronbach's alpha coefficients of 0.70 or higher were considered acceptable. Composite reliability was also examined as part of the measurement-model assessment.

Confirmatory factor analysis was conducted in Amos to evaluate the measurement model. Construct validity was assessed by examining standardized factor loadings, composite reliability, average variance extracted, and convergent and discriminant validity. Structural equation modeling was subsequently used to test the hypothesized direct and indirect relationships among the constructs. Indirect effects were examined using [insert number] bootstrap resamples and 95% bias-corrected confidence intervals. A mediation effect was considered statistically significant when its confidence interval did not include zero.

Model fit was evaluated using the chi-square statistic (χ^2^), degrees of freedom, chi-square-to-degrees-of-freedom ratio (χ^2^/df), comparative fit index (CFI), Tucker–Lewis index (TLI), root mean square error of approximation (RMSEA) with its 90% confidence interval, and standardized root mean square residual (SRMR). Values of χ^2^/df below 5.00, CFI and TLI of at least 0.90, and RMSEA and SRMR of 0.08 or lower were considered indicative of acceptable fit. These criteria were interpreted collectively rather than as absolute cut-off rules (Hu and Bentler, 1999; Marsh et al., 2004).

Because all constructs were measured through self-report questionnaires collected at a single time point, procedural measures were used to reduce potential common method variance. These included anonymous participation, confidentiality assurances, clear questionnaire instructions, and separation of the predictor and outcome measures within the questionnaire. Negatively worded items were reverse-scored before analysis. However, these procedures were not considered sufficient to eliminate common method variance completely (Podsakoff et al., 2003).

### 2.4 Ethical considerations

Ethical approval was obtained from Mwalimu Nyerere Memorial Academy and St. Augustine University of Tanzania. Permission to conduct the study was also obtained from the relevant authorities at both participating institutions. All participants provided informed consent before taking part in the study.

## 3 Results

### 3.1 Participant characteristics

Following data screening, 22 of the 487 returned questionnaires were excluded because at least 95% of the substantive item responses used the same response category. The final analytic sample therefore comprised 465 second- and third-year pre-service teachers, including 252 participants from Mwalimu Nyerere Memorial Academy, Kivukoni Campus, Dar es Salaam, and 213 from St. Augustine University of Tanzania, Mwanza. This represented a valid-response rate of 95.5%.

The sample included 259 male participants (55.7%) and 206 female participants (44.3%). Most participants were aged 21–25 years (*n* = 334, 71.8%), followed by those aged 26–30 years (*n* = 70, 15.1%), 16–20 years (*n* = 24, 5.2%), 31–35 years (*n* = 22, 4.7%), and 36 years or older (*n* = 15, 3.2%). Regarding year of study, 238 participants (51.2%) were in their second year and 227 (48.8%) were in their third year. Table 1 presents the participants' demographic characteristics.

**Table 1:** Demographic characteristics of pre-service teachers respondents (*n* = 465).

Demographic variables	Descriptions	No of respondents	Percentage (%)
Gender	Male	259	55.7
	Female	206	44.3
Age	16–20	24	5.4
	21–25	334	71.8
	26–30	70	15.1
	31–35	22	4.7
	36+	15	3.2
Year of study	2nd Year	238	51.2
	3rd Year	227	48.8
Total participants		**465**	**100%**

Bold values indicate the total number and percentage of participants.

### 3.2 Measurement model

Confirmatory Factor Analysis (CFA) was conducted using AMOS Version 26 to assess the measurement properties of the five study constructs: Quality of University Curriculum (QUC), Faculty Support and Modeling (FAS), Peer Support (PEST), Pre-service Teachers' Beliefs in Social-emotional Learning (PTBSEL), and Pre-service Teachers' Social-emotional Competence (PTSEC). Following established CFA procedures, items with weak standardized factor loadings were removed to improve the measurement model (Hair et al., 2009; Meyers et al., 2006). After item refinement, PTSEC retained 6 of its original 10 items, QUC retained 5 of its original 9 items, while FAS, PEST, and PTBSEL retained all their original items. The retained standardized factor loadings ranged from 0.56 to 0.83, which is considered acceptable for applied social science research (Hair et al., 2009).

The final full measurement model demonstrated acceptable overall fit: CMIN/DF = 2.052, CFI = 0.947, RMSEA = 0.048, SRMR = 0.0373, NFI = 0.949, TLI = 0.942, and PClose = 0.794. These values met commonly recommended thresholds for model fit, including CMIN/DF below 3.0, CFI, NFI, and TLI above 0.90, RMSEA below 0.08, SRMR below 0.09, and PClose above 0.05 (Bentler and Bonett, 1980; Hatcher, 1994; Hair et al., 2009; Hu and Bentler, 1999; Karimi and Meyer, 2014; Meyers et al., 2006). Composite reliability values ranged from 0.864 to 0.885, exceeding the recommended threshold of 0.70, while Average Variance Extracted values ranged from 0.559 to 0.644, exceeding the recommended minimum value of 0.50 and indicating acceptable convergent validity (Fornell and Larcker, 1981; Hair et al., 2009).

However, the deletion of several items, particularly from PTSEC and QUC, suggests that some adapted measures may not have fully captured the intended constructs in the Tanzanian teacher education context. This may reflect contextual differences in how pre-service teachers understand social-emotional competence and curriculum quality. Therefore, although the refined model achieved acceptable statistical fit, the results should be interpreted with caution. Future studies should conduct pilot testing, cultural validation, cognitive interviewing, and further construct refinement before using these instruments in broader Tanzanian or Sub-Saharan African contexts (Fornell and Larcker, 1981; Hair et al., 2009).

In addition, all study variables were measured using self-report Likert-scale questionnaires collected from the same respondents at a single point in time. This design increases the possibility of common method variance, social desirability bias, and perceptual consistency effects (Podsakoff et al., 2003). Although anonymity and voluntary participation were used to reduce response bias, these procedural controls cannot fully eliminate common method bias. The high correlations among constructs and the large explained variance in the structural model may also suggest possible construct overlap or model over fitting. Future research should therefore use multiple data sources, such as faculty ratings, classroom observations, practicum assessments, interviews, or longitudinal measures, to strengthen construct validity and reduce common method bias (Podsakoff et al., 2003; Hair et al., 2009).

**Figure 1:**
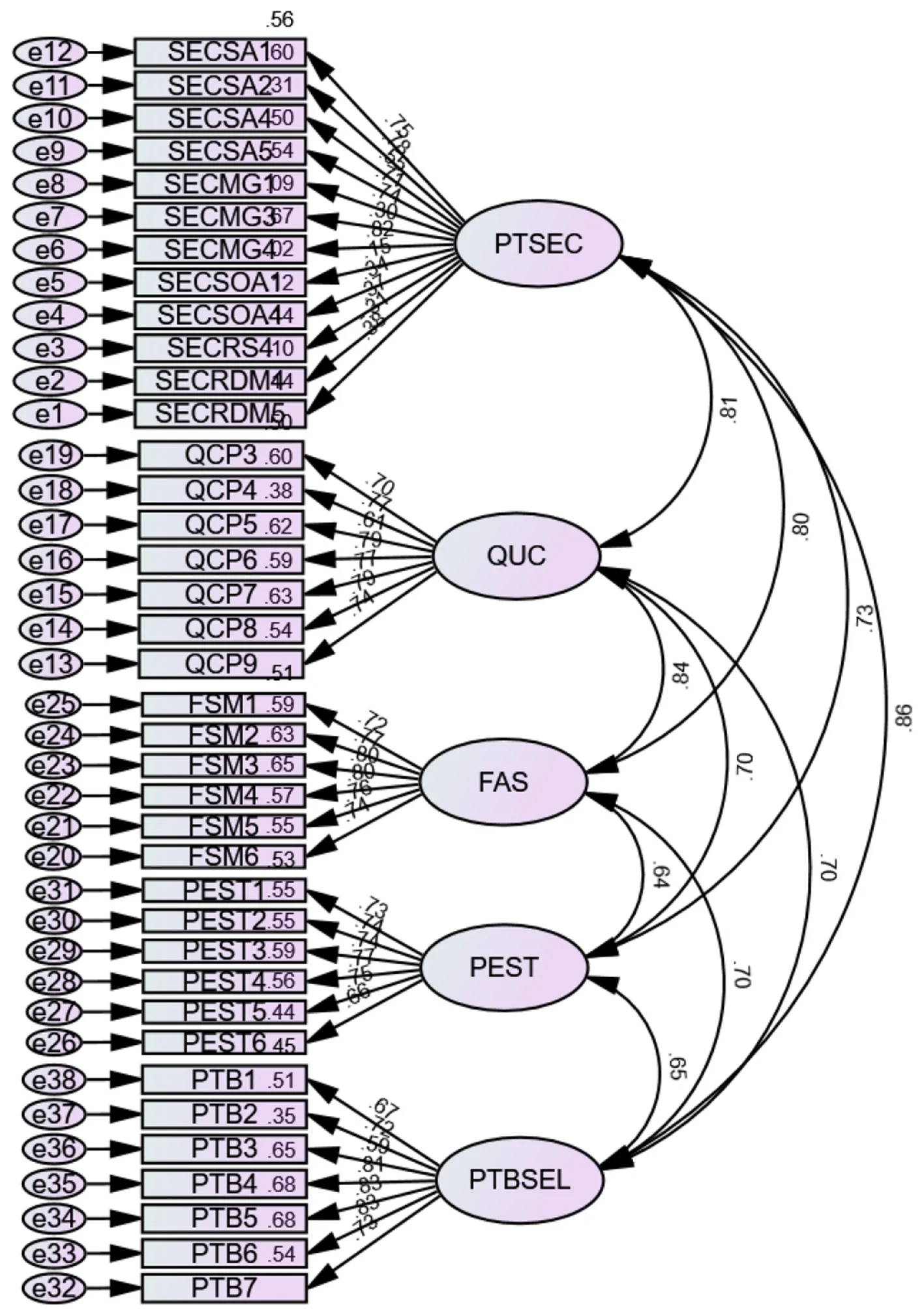
The full measurement model of pre-service teachers' social-emotional competence.

The measurement model results with its standardized loading factors in which the lowest factor loading was 0.56, while the highest factor loading was 0.83 are presented in the following Figure 1.

After running the above model the Confirmatory Factor Analysis (CFA) results indicated excellent Confirmatory Fit Index (CFI) = 0.947, excellent chi-square (CMIN/DF) = 2.052, excellent Root Mean Square Error of Approximation (RMSEA) = 0.048, excellent Standardized Root Mean Square Residual (SRMR) = 0.0373, excellent Normed Fit Index (NFI) = 0.949, excellent (PClose) = 0.794, and excellent Tucker-Lewis Index (TLI) = 0.942. Table 2 presents the results of the model assessment from confirmatory factor analysis.

**Table 2:** The summary of the full model assessment results from CFAs.

Fit indices	Sources	Recommended values	Results obtained
χ^2^	Meyers et al. (2006)	*P*-value >0.05	0.000
CMIN/DF	Hair et al. (2009)	< 3.0 Good; < 5.0 sometimes permissible	2.052
CFI	Hatcher (1994)	>0.90	0.947
RMSEA	Karimi and Meyer (2014)	< 0.08	0.048
SRMR	Hair et al. (2009)	< 0.09	0.0373
NFI	Bentler and Bonett (1980)	>0.90	0.949
TLI	Hu and Bentler (1999)	>0.90	0.942
PClose	Hair et al. (2009)	>0.05	0.794

The above fit indices obtained excellent results. On that regard it was conclude that the measurement instrument model met the expected standards.

Applying AMOS version 26 to examine composite reliability (CR) of the measurement models for each construct and average variance extracted (AVE), the results are displayed in Table 3. CR ranges from 0.864 to 0.885 and is below the recommended level of 0.5. AVE may be a more conservative estimate of the validity of the measurement model and “on the basis adequate, even though more than 0.5 of the variance is due to error” (Meyers et al., 2006). As the CR is well above the recommended level, the internal reliability of the measurement items is acceptable.

**Table 3:** Results of confirmatory factor analysis.

Variables	Composite reliability (CR)	Average variance extracted (AVE)
Pre-service teachers' social-emotional competence	0.870	0.572
Quality of university curriculum	0.882	0.599
Faculty support	0.885	0.606
Peer support	0.864	0.559
Belief on social-emotional learning	0.879	0.644

There are several methods to assess convergent validity; however, in this study, the Average Variance Extracted (AVE) proposed by Fornell and Larcker (1981) was used. AVE measures the amount of variance captured by a construct in relation to the amount of variance due to measurement error. An AVE value of 0.5 or higher indicates adequate convergent validity, as it means that the construct explains more than half of the variance of its indicators. Based on those criteria, CRs surpass the acceptable criteria/range; in this regard, the convergent validity of the constructs in the measurement model was considered more adequate. See Table 3 for detailed information.

With regard to the discriminant validity of all latent variables, the bolded values in the diagonal are the square root of AVE while the other values with three stars in the table are just the correlation between the constructs. For example **0.756** is a square root of AVE for PTSEC and 0.702 is the correlation between pre-service teachers social-emotional competence (PTSEC) and quality of university curriculum (QUC) while on the other hand ^***^signify that the correlation are significant. Based on the criteria for assessing discriminant validity, the square root of AVE for each latent variable should be greater than all values within the same column and the row. Given that criteria the AVE for each latent variable as presented in the above table are all significantly higher than the square correlations between construct. This provides justification that all five latent variables had good discrimination validity. The results of the convergent and discriminant validity assessment are presented in Table 4.

**Table 4:** Convergent and discriminant validities of measures.

Construct	Mean	Std.	1	2	3	4	5
PTSEC	3.0933	1.00980	**0.756**				
QUC	2.9072	1.13738	0.702^***^	**0.774**			
FAS	2.8587	1.01972	0.681^***^	0.758^***^	**0.778**		
PEST	2.7772	1.03428	0.642^***^	0.686^***^	0.625^***^	**0.748**	
PTBSEL	3.1629	1.00767	0.627^***^	0.683^***^	0.603^***^	0.642^***^	**0.803**

Bold diagonal values indicate the square root of the average variance extracted (AVE) for each construct. ^***^p < 0.001.

On other hand, the Heterotrait-Monotrait Ratio (HTMT) approach was used in assessing the discriminant validity in AMOS version 26. Henseler et al. (2015) proposed the Heterotrait-Monotrait ratio of correlations (HTMT) as a new approach for assessing discriminant validity. The HTMT method involves comparing the ratio of between-construct correlations to within-construct correlations. The criteria for assessing discriminant validity are that discriminant validity is established if the HTMT value is below a certain threshold. Generally, a threshold value of 0.85 or 0.90 is used. If HTMT is below this threshold, it suggests that the constructs are discriminant. Based on these criteria the all values obtained were below the acceptable threshold range which provides evidence of the presence of discriminant validity. This information can be obtained in Table 5 that presents the HTMT analysis for discriminant validity.

**Table 5:** HTMT analysis for discriminant validity.

Construct	PTSEC	QUC	FAS	PEST	PTBSEL
PTSEC					
QUC	0.799				
FAS	0.790	0.802			
PEST	0.722	0.684	0.615		
PTBSEL	0.821	0.679	0.690	0.636	

NB, The Heterotrait-Monotrait (HTMT) ratio of correlations for each construct is below the threshold of 0.850 or 0.900, which confirms the presence of discriminant validity (Gaskin et al., 2019).

To evaluate the internal consistence of the scales, the SPSS 29.0 was used to calculate Chronbach's alpha coefficient. The results indicated that the instruments had acceptable Chronbach's alpha coefficient. In this regard it was concluded that the measurement model met the desired standards of reliability and validity as indicated in Table 6.

**Table 6:** Reliability test of each scale.

Reliability statistic	Pre-service teachers social-emotional competence scale	Quality of university curriculum	Faculty support	Peer support	Belief on social-emotional learning
Chronbach's alpha	0.879 (no items 25)	0.903 (no items 9)	0.892 (no Items 6)	0.874 (No items 6)	0.890 (no items 7)

### 3.3 Descriptive statistics and correlation analysis (No. 465)

In this part a Pearson correlation analysis was conducted to examine the relationships between several variables: pre-service teachers' social-emotional competence (PTSEC), quality of the university curriculum (QUC), faculty support (FAS), peer support (PEST), and pre-service teachers belief on social-emotional learning (PTBSEL). Furthermore, the nature of strength of the relationship between these variables was examined. The detailed results have been presented in the Table 7. The correlations matrix.

**Table 7:** The correlation matrix of the variables (*n* = 465).

Variable	Mean	Std.	1	2	3	4	5
PTSEC	3.992	0.758	1				
QUC	3.946	0.773	0.643^**^	1			
FAS	3.945	0.829	0.571^**^	0.758^**^	1		
PEST	2.884	0.779	0.618^**^	0.712^**^	0.689^**^	1	
PTBSEL	4.027	0.792	0.580^**^	0.652^**^	0.631^**^	0.774^**^	1

^**^Correlation is significant at the 0.01 level (2-tailed).

A Pearson product-moment correlation was conducted to examine the relationships among peer support, quality of university curriculum, faculty support, pre-service teachers' social-emotional competence, and pre-service teachers' belief in SEL. Results indicated that peer support was positively and significantly correlated with university curriculum (*r* = 0.64, *p* < 0.001), faculty support (*r* = 0.57, *p* < 0.001), social-emotional competence (*r* = 0.62, *p* < 0.001), and belief in SEL (*r* = 0.58, *p* < 0.001). Similarly, university curriculum was strongly and positively associated with faculty support (*r* = 0.76, *p* < 0.001), social-emotional competence (*r* = 0.71, *p* < 0.001), and belief in SEL (*r* = 0.65, *p* < 0.001).

Faculty support also showed significant positive correlations with social-emotional competence (*r* = 0.69, *p* < 0.001) and belief in SEL (*r* = 0.63, *p* < 0.001). Furthermore, pre-service teachers' social-emotional competence was highly correlated with their belief in SEL (*r* = 0.77, *p* < 0.001). All correlations were statistically significant at the 0.01 level (two-tailed), suggesting strong positive relationships among all variables measured.

### 3.4 Testing the hypothesized indirect associations

A structural equation model was specified to examine the hypothesized direct and indirect associations among perceived quality of the university curriculum (QUC), faculty support (FAS), peer support (PEST), pre-service teachers' beliefs in social-emotional learning (PTBSEL), and pre-service teachers' social-emotional competence (PTSEC). QUC, FAS, and PEST were specified as exogenous variables, PTBSEL as the proposed statistical mediator, and PTSEC as the outcome variable.

**Figure 2:**
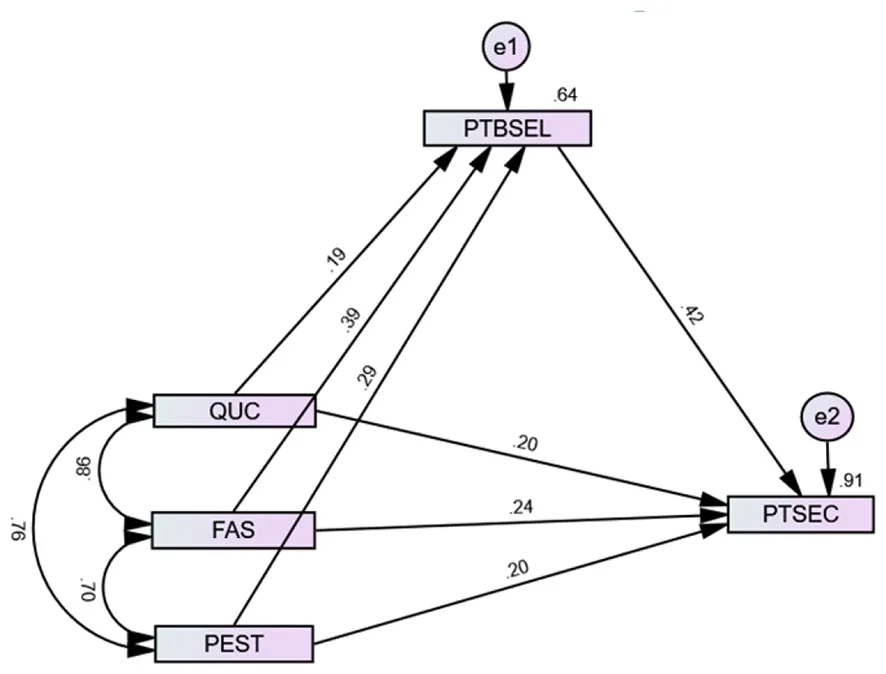
Results of the hypothesized indirect-association model.

The model demonstrated generally acceptable fit to the data: RMSEA = 0.046, CFI = 0.964, TLI = 0.956, SRMR = 0.093, Pclose = 0.879, CMIN/DF = 1.969, and NFI = 0.930. Although the SRMR was slightly above the commonly applied threshold of 0.08, the remaining fit indices indicated that the hypothesized model provided an acceptable representation of the observed data. Figure 2 presents the standardized path coefficients for the hypothesized model.

The statistical significance of the indirect associations was examined using bootstrap resampling. Following Zhao et al. (2010), 2,000 bootstrap samples and 95% confidence intervals were generated in AMOS version 26. This procedure was used to examine whether QUC, FAS, and PEST were indirectly associated with PTSEC through PTBSEL. The results are presented in Table 8.

**Table 8:** Standardized direct, indirect, and total associations of QUC, FAS, and PEST with PTSEC.

Predictor	Association type	β	*p*	Interpretation
QUC → PTSEC	Direct	0.200	< 0.001	Direct association remained significant
	Indirect through PTBSEL	0.079	< 0.001	Significant indirect association
	Total	0.278	< 0.001	Significant total association
FAS → PTSEC	Direct	0.240	< 0.001	Direct association remained significant
	Indirect through PTBSEL	0.162	< 0.001	Significant indirect association
	Total	0.401	< 0.001	Significant total association
PEST → PTSEC	Direct	0.197	< 0.001	Direct association remained significant
	Indirect through PTBSEL	0.121	< 0.001	Significant indirect association
	Total	0.318	< 0.001	Significant total association

QUC, quality of university curriculum; FAS, faculty support; PEST, peer support; PTBSEL, pre-service teachers' beliefs in social-emotional learning; PTSEC, pre-service teachers' social-emotional competence. The indirect associations represent statistical mediation within the hypothesized cross-sectional model and should not be interpreted as evidence of a temporal or causal process. Minor differences between the sum of the reported direct and indirect coefficients and the total coefficients may result from rounding of the standardized estimates.

The first hypothesis proposed that QUC, FAS, and PEST would be positively associated with PTBSEL. The results supported this hypothesis. QUC was positively associated with PTBSEL (β = 0.19, *p* = 0.002), as were FAS (β = 0.39, *p* < 0.001) and PEST (β = 0.29, *p* < 0.001). Together, these variables accounted for 63.9% of the variance in PTBSEL (*R*^2^ = 0.639).

The second hypothesis proposed that QUC, FAS, and PEST would have positive direct associations with PTSEC. The results also supported this hypothesis. QUC (β = 0.20, *p* < 0.001), FAS (β = 0.24, *p* < 0.001), and PEST (β = 0.20, *p* < 0.001) were each positively and significantly associated with PTSEC after PTBSEL was included in the model.

The third hypothesis proposed that PTBSEL would be positively associated with PTSEC. This hypothesis was supported, as PTBSEL showed a positive and statistically significant association with PTSEC (β = 0.42, *p* < 0.001). Collectively, QUC, FAS, PEST, and PTBSEL accounted for 90.6% of the variance in PTSEC (*R*^2^ = 0.906).

The fourth hypothesis proposed that QUC, FAS, and PEST would have significant indirect associations with PTSEC through PTBSEL. The results supported the hypothesized indirect associations. QUC had a significant indirect association with PTSEC through PTBSEL (β = 0.079, *p* < 0.001). Significant indirect associations were also found for FAS (β = 0.162, *p* < 0.001) and PEST (β = 0.121, *p* < 0.001).

Because the corresponding direct associations between QUC, FAS, PEST, and PTSEC remained statistically significant after PTBSEL was included, the results were consistent with partial statistical mediation. In other words, PTBSEL accounted for part, but not all, of the associations between the three environmental variables and PTSEC. Given the cross-sectional design, however, these findings should be understood as statistical indirect associations consistent with the hypothesized mediation model rather than evidence that PTBSEL causally transmits the effects of QUC, FAS, or PEST to PTSEC.

Considering the direct and indirect pathways together, FAS showed the largest total association with PTSEC (β = 0.401), followed by PEST (β = 0.318) and QUC (β = 0.278). These comparisons describe the relative magnitudes of the associations observed within the specified model and should not be interpreted as evidence that one factor has a stronger causal influence than another.

The exogenous variables were also strongly intercorrelated. QUC was positively correlated with FAS (*r* = 0.86, *p* < 0.001) and PEST (*r* = 0.76, *p* < 0.001), while FAS was positively correlated with PEST (*r* = 0.70, *p* < 0.001). Because the correlation between QUC and FAS was particularly high, the individual path coefficients should be interpreted cautiously, as the variables may represent closely related aspects of the institutional environment.

## 4 Discussion

This study examined the relationships between perceived quality of the university curriculum (QUC), faculty support (FAS), peer support (PEST), pre-service teachers' beliefs in social-emotional learning (PTBSEL), and pre-service teachers' social-emotional competence (PTSEC) in Tanzania. QUC, FAS, and PEST were positively associated with both PTBSEL and PTSEC, while PTBSEL was positively associated with PTSEC. PTBSEL also showed significant partial indirect associations between each environmental factor and PTSEC. These findings are broadly consistent with Social Cognitive Theory (SCT), which proposes that competence develops through interactions among environmental conditions, personal cognitive factors, and behavioral capabilities (Bandura, 1986). However, because the data were cross-sectional and self-reported, the findings indicate statistical associations consistent with the proposed model rather than causal relationships.

### 4.1 Curriculum quality and social-emotional competence

QUC was positively associated with PTBSEL (β = 0.19, *p* = 0.002) and PTSEC (β = 0.20, *p* < 0.001). It also showed a significant indirect association with PTSEC through PTBSEL (β = 0.08, *p* < 0.001), resulting in a total standardized association of β = 0.28. These findings suggest that pre-service teachers who perceived their curricula as providing relevant opportunities for social-emotional learning also reported stronger beliefs in SEL and higher levels of SEC.

The finding is consistent with research indicating that SEC can be developed through explicit instruction, guided reflection, collaborative learning, role-play, simulations, and opportunities to connect theoretical knowledge with teaching practice (Darwich et al., 2025; Monroy Correa and Manzanal Martínez, 2025). Tripon (2023) similarly argues that emotional intelligence supports adaptability, communication, teamwork, decision-making, and effective functioning in changing professional environments. Although Tripon's study focused on STEM professionals rather than Tanzanian pre-service teachers, it supports the broader proposition that professional preparation should develop emotional and interpersonal competencies alongside technical and disciplinary knowledge.

The present findings do not demonstrate that curriculum quality caused stronger PTSEC. Pre-service teachers with stronger SEC may also have evaluated their curricula more positively. Longitudinal and intervention studies are therefore required to determine whether changes in curriculum experiences precede changes in beliefs and competence.

### 4.2 Faculty support and social-emotional competence

FAS was positively associated with PTBSEL (β = 0.39, *p* < 0.001) and PTSEC (β = 0.24, *p* < 0.001). Its indirect association with PTSEC through PTBSEL was also significant (β = 0.16, *p* < 0.001), resulting in the largest total standardized association among the three environmental factors (β = 0.40).

From an SCT perspective, faculty members may influence learning through modeling, feedback, encouragement, and social persuasion (Bandura, 1986). Teacher educators who demonstrate emotional regulation, empathy, fairness, respectful communication, and constructive responses to challenging situations provide observable examples from which pre-service teachers may learn. Hooper and Johnson (2025) similarly emphasize the role of teacher educators in modeling SEL, embedding it within teacher-preparation experiences, and creating supportive learning environments.

Nevertheless, this study measured participants' perceptions of faculty support rather than directly observing faculty practices. The finding therefore indicates an association between perceived support and PTSEC, not evidence that particular faculty behaviors caused competence development. Moreover, FAS was strongly correlated with QUC (r = 0.86), suggesting that participants may not have clearly distinguished the support provided by faculty members from the quality of the curriculum they delivered. The apparent strength of the FAS pathway should consequently be interpreted cautiously.

### 4.3 Peer support and social-emotional competence

PEST was positively associated with PTBSEL (β = 0.29, *p* < 0.001) and PTSEC (β = 0.20, *p* < 0.001). It also showed a significant indirect association with PTSEC through PTBSEL (β = 0.12, *p* < 0.001), producing a total standardized association of β = 0.32. Supportive peer relationships may provide opportunities to practice cooperation, empathy, perspective-taking, communication, emotional expression, and constructive conflict resolution.

Peer support is particularly relevant in teacher education because pre-service teachers frequently participate in group assignments, peer observation, microteaching, collaborative reflection, and shared practicum experiences. Experiential and collaborative learning activities may allow them to practice social-emotional skills while receiving feedback and emotional support from others (Darwich et al., 2025; Monroy Correa and Manzanal Martínez, 2025).

The finding is also relevant to culturally grounded understandings of SEC in Tanzania. Research conducted in Tanzania has identified locally valued competencies such as respect, responsibility, cooperation, attentive listening, and socially appropriate behavior (Jukes et al., 2021). Tandika et al. (2025) similarly demonstrate the importance of contextually relevant understandings of social-emotional competence. Peer relationships may therefore support SEC through both individual skill development and participation in culturally valued forms of cooperation and mutual responsibility.

### 4.4 Indirect role of beliefs in SEL

PTBSEL was positively associated with PTSEC (β = 0.42, *p* < 0.001) and partially mediated the relationships of QUC, FAS, and PEST with PTSEC. The findings suggest two complementary pathways. First, curriculum, faculty, and peer environments may be directly associated with SEC by providing opportunities for modeling, interaction, practice, feedback, and reflection. Second, these environmental conditions may be indirectly associated with SEC through stronger beliefs in the relevance, value, and applicability of SEL.

Pre-service teachers may engage more actively with social-emotional learning opportunities when they believe that SEL is important for teaching and feel comfortable developing and applying the relevant skills. Choquette et al. (2024) similarly found that pre-service teachers' comfort-related SEL beliefs were associated with aspects of their relationship skills.

However, PTBSEL should not be treated as equivalent to self-efficacy. The construct incorporates broader orientations involving comfort, confidence, commitment, and perceived value. These dimensions may operate differently, and the present study cannot determine which specific belief dimension accounted for the observed indirect associations.

The mediation findings should also be interpreted cautiously. Mediation implies a temporal process, but the environmental variables, beliefs, and PTSEC were measured concurrently. Cross-sectional mediation estimates may not accurately represent the corresponding longitudinal process (Maxwell and Cole, 2007). The results therefore provide evidence of indirect statistical associations consistent with mediation, rather than proof that environmental support first changed beliefs and that those beliefs subsequently caused stronger SEC.

### 4.5 Theoretical interpretation and contribution

The findings largely support relationships anticipated by Social Cognitive Theory and therefore do not constitute a fundamental extension or challenge to the theory. Rather, the study offers a context-specific refinement of how environmental and cognitive factors may operate in pre-service teacher education. Social Cognitive Theory proposes that learning and competence develop through reciprocal interactions among environmental conditions, personal cognitive factors, and behavior or capability development (Bandura, 1986).

First, the findings differentiate the environmental component of SCT into three related but conceptually distinct pathways. Curriculum quality represents formal and structured opportunities for acquiring, practicing, and reflecting on social-emotional skills. Faculty support represents modeling, guidance, feedback, and social persuasion, whereas peer support represents collaborative learning, vicarious experience, emotional reinforcement, and horizontal social relationships. This distinction provides a more specific account of the teacher-education environment than treating environmental support as a single undifferentiated influence. Modeling, feedback, mastery experiences, and social persuasion are recognized mechanisms through which educational environments may shape competence-related beliefs and behavior (Bandura, 1986; Täschner et al., 2025).

Second, the findings identify beliefs in SEL as a possible domain-specific cognitive mechanism connecting educational environments with social-emotional competence. Curriculum, faculty, and peer experiences may not automatically translate into competence because pre-service teachers differ in the extent to which they value SEL, feel comfortable engaging with it, and regard it as relevant to their future professional practice. Previous research similarly indicates that pre-service teachers' beliefs and comfort regarding SEL are associated with aspects of their relationship competence (Choquette et al., 2024).

Third, the partial indirect associations suggest that environmental experiences and personal beliefs operate through complementary pathways. Curriculum quality, faculty support, and peer support were directly associated with PTSEC and indirectly associated with it through PTBSEL. This pattern suggests that supportive environments may provide opportunities for modeling, interaction, feedback, and practice, while positive beliefs may strengthen pre-service teachers' willingness to engage with and apply those opportunities. However, PTBSEL should not be treated as equivalent to self-efficacy because it includes broader dimensions such as comfort, commitment, confidence, and perceived value (Brackett et al., 2012; Choquette et al., 2024).

The partial rather than full mediation also indicates that positive beliefs cannot substitute for appropriate curriculum experiences, faculty guidance, and peer interaction. Instead, institutional supports and cognitive orientations appear to function as mutually reinforcing components of competence development. This interpretation refines a simple environment-to-competence pathway by identifying beliefs as a possible mechanism through which environmental opportunities become personally meaningful.

Nevertheless, the present study tested only a unidirectional representation of SCT. Reciprocal determinism permits alternative explanations: pre-service teachers with stronger social-emotional competence may develop more positive beliefs about SEL or may evaluate their curriculum, faculty, and peers more favorably (Bandura, 1986). Moreover, because the variables were measured concurrently, the indirect effects do not establish temporal or causal mediation. Cross-sectional mediation estimates may differ from the corresponding longitudinal processes (Maxwell and Cole, 2007).

Overall, the study makes a modest theoretical contribution by specifying distinct environmental pathways and identifying beliefs in SEL as a potential cognitive mechanism within Tanzanian pre-service teacher education. Its contribution is therefore primarily contextual and explanatory rather than the development of a new theory. Longitudinal, cross-lagged, and intervention studies are needed to examine temporal ordering, reciprocal relationships, and the distinct functions of different SEL-belief dimensions.

### 4.6 Tanzanian teacher-education context

The findings should be understood within the organization of teacher education in Tanzania. Current Tanzania Commission for Universities guidelines describe bachelor's teacher-education programmes as three-year programmes combining academic subject preparation with education courses. The education component includes educational psychology, professional ethics, curriculum and teaching, inclusive education, professional communication, teaching methods, and practical training. These areas provide possible entry points for developing self-awareness, self-management, empathy, relationship skills, and responsible decision-making (Tanzania Commission for Universities, 2024).

However, the presence of related courses does not demonstrate that SEL is systematically integrated into teacher education. The absence of an explicitly identified SEL strand in the current guidelines suggests that SEC-related preparation may be distributed across courses and practicum activities rather than organized as a coherent programme component. Because these guidelines were issued after the present data were collected, they represent the current policy and reform context rather than necessarily describing the precise curriculum experienced by every participant.

Direct evidence concerning systematic SEL integration in Tanzanian university-based teacher preparation remains limited. Existing Tanzanian studies have focused primarily on children, practicing teachers, early childhood education, and inclusive primary-school settings. Shaffy and Ndijuye (2025), for example, found that socio-emotional intelligence was associated with the pedagogical competence of Tanzanian pre-primary teachers. Such findings support the professional relevance of emotional and interpersonal competencies but do not establish that SEL is consistently incorporated into university teacher-education programmes.

### 4.7 Cultural and institutional considerations

International frameworks such as CASEL provide useful organizing concepts, but their application should be informed by local cultural understandings. Tanzanian research shows that community-defined SEC includes respect, responsibility, cooperation, attentiveness, and socially appropriate conduct, which may not correspond perfectly with externally developed frameworks (Jukes et al., 2021). Accordingly, SEC integration should involve consultation with pre-service teachers, teacher educators, schools, and communities rather than relying only on the linguistic adaptation of imported curricula and instruments.

Institutional conditions may also shape implementation. Tanzania's Education Sector Development Plan identifies infrastructure shortages in teacher-education institutions, inadequate resources, insufficient continuing professional development for tutors and lecturers, limited access to technology and internet connectivity, and gaps between policy formulation and implementation (United Republic of Tanzania, 2025). These constraints may affect institutions' ability to introduce additional modules, prepare faculty members, support practicum supervision, and maintain mentoring or peer-learning structures.

### 4.8 Preliminary implementation framework for social-emotional competence in teacher education

Building on the SEM findings, theoretical interpretation, and Tanzanian context, this study proposes a Preliminary Implementation Framework for Social-Emotional Competence in Teacher Education (IF-SEC-TE). The framework translates the study's empirically informed components and literature-derived implementation phases into a structure for future piloting and evaluation. It is not presented as an empirically validated intervention, and none of its four phases emerged directly from the survey data.

The SEM findings informed the selection and positioning of the framework's core components. Curriculum quality, faculty support, and peer support are positioned as environmental influences; PTBSEL is positioned as a personal cognitive mechanism; and PTSEC is positioned as the intended developmental outcome. The four phases and their sequence were derived from literature on explicit SEL instruction, teacher-educator modeling, experiential learning, reflective practice, practicum, and continuing professional development (Darwich et al., 2025; Hooper and Johnson, 2025; Monroy Correa and Manzanal Martínez, 2025).

The sequence follows a pedagogical progression. Phase 1, Building Foundations and Awareness, introduces SEL concepts through explicit instruction, reflective discussion, culturally relevant examples, and faculty modeling. Phase 2, Skills Building and Practical Integration, provides opportunities to practice self-awareness, self-management, empathy, communication, and relationship skills through microteaching, simulation, role-play, peer learning, and formative feedback. Phase 3, Teaching Practicum and Field Experience, supports the application of SEC in authentic classrooms, including classroom management, teacher–student relationships, conflict resolution, and professional decision-making. Phase 4, Consolidation and Long-Term Development, sustains SEC through mentoring, professional learning communities, peer networks, and continuing development in teacher wellbeing, resilience, and leadership. SEC is also relevant to pre-service teachers' occupational health and personal wellbeing (Braun and Hooper, 2024).

**Figure 3:**
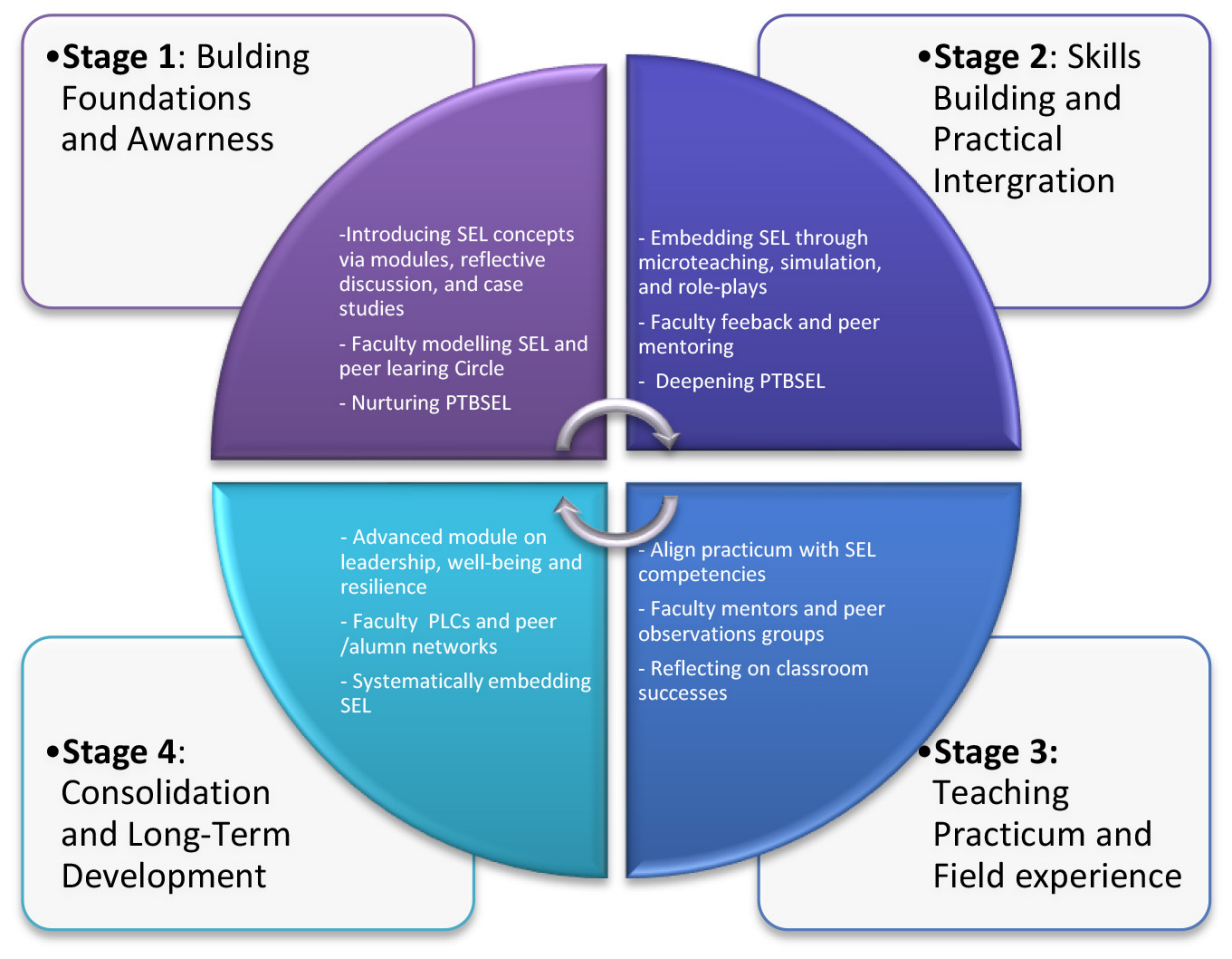
Preliminary Implementation Framework for developing Social-Emotional Competence in Teacher Education (IF-SEC-TE). Source: Author's own elaboration.

Across the phases, curriculum quality, faculty support, peer support, and beliefs in SEL operate as interconnected components. However, the present study did not test the feasibility, sequence, or effectiveness of the framework. The IF-SEC-TE should therefore be regarded as a preliminary and testable proposal for future longitudinal, intervention, and implementation research. Figure 3 shows Preliminary Implementation Framework for developing Social-Emotional Competence in Teacher Education (IF-SEC-TE).

### 4.9 Practical implications

The empirical findings indicate that perceived curriculum quality, faculty support, peer support, and beliefs in SEL are associated with self-reported SEC. They do not demonstrate the effectiveness of any specific programme. Nevertheless, the findings and prior literature suggest that Tanzanian teacher-education institutions could consider mapping SEC across existing courses, including educational psychology, professional ethics, curriculum and teaching, inclusive education, professional communication, teaching methods, and practicum.

Embedding SEC within existing courses may be more feasible than relying exclusively on additional stand-alone modules, particularly in institutions facing resource and staffing constraints. Faculty modeling, microteaching, role-play, reflective journals, peer mentoring, and practicum-based feedback could provide opportunities for supported practice. These proposals remain theoretically and literature-informed recommendations until their effectiveness is evaluated empirically.

### 4.10 Limitations and directions for future research

Several limitations should be considered. First, the cross-sectional design does not establish temporal order or causality. Second, all variables were measured through self-report questionnaires completed by the same participants, which may have inflated the associations through common method variance, social desirability, or perceptual consistency (Podsakoff et al., 2003).

Third, the model explained 90.6% of the variance in PTSEC, which is unusually high in social-science research. Although this may indicate that the selected variables are closely related to PTSEC, it may also reflect construct overlap or shared method variance. The strong correlations among QUC, FAS, and PEST—particularly the QUC–FAS correlation of *r* = 0.86—also suggest that their unique contributions should be interpreted cautiously.

Fourth, the study included participants from only two institutions, limiting generalisability across Tanzania's diverse teacher-education system. In addition, the deletion of several items during measurement-model refinement may have reduced the content coverage of some constructs.

Future studies should employ longitudinal and cross-lagged designs to examine temporal and reciprocal relationships among environmental support, beliefs, and SEC. Multi-source assessments incorporating faculty ratings, peer evaluations, classroom observations, practicum assessments, and qualitative interviews would reduce reliance on self-report. Further cultural validation and cognitive interviewing are also needed to establish how Tanzanian pre-service teachers interpret SEC and SEL-belief items. Finally, pilot and quasi-experimental studies should evaluate whether curriculum integration, faculty development, peer-learning arrangements, and practicum-based interventions produce sustained improvements in SEC, professional wellbeing, and classroom practice.

## 5 Conclusion

This study examined how perceived university curriculum quality, faculty support, and peer support were associated with social-emotional competence among pre-service teachers in Tanzania, and whether pre-service teachers' beliefs in social-emotional learning accounted for part of these relationships. All three environmental factors were positively associated with beliefs in SEL and social-emotional competence. Beliefs in SEL were also positively associated with competence and showed significant indirect associations between each environmental factor and social-emotional competence. These findings suggest that SEC development in teacher education is related to both supportive institutional environments and pre-service teachers' cognitive orientations toward SEL.

The study provides a modest theoretical refinement of Social Cognitive Theory by distinguishing curriculum, faculty, and peer support as related but conceptually different environmental pathways and by identifying beliefs in SEL as a possible domain-specific cognitive mechanism. Its principal contribution is contextual and explanatory: it extends evidence on SEC to Tanzanian university-based teacher education, where systematic SEL integration remains limited and must be considered in relation to local cultural understandings and institutional conditions.

The findings support greater attention to SEC within existing teacher-education courses, faculty practices, peer-learning activities, and teaching practicum. The proposed Preliminary Implementation Framework for Social-Emotional Competence in Teacher Education translates these findings and relevant literature into a structure for future piloting rather than a validated intervention. Because the study relied on cross-sectional self-report data from two institutions, it cannot establish temporal or causal relationships. Longitudinal, multi-source, and intervention studies are therefore required to test the proposed mechanisms, evaluate the implementation framework, and determine whether culturally responsive SEL preparation produces sustained improvements in pre-service teachers' competence, wellbeing, and classroom practice.

## Data Availability

The raw data supporting the conclusions of this article will be made available by the authors, without undue reservation.
